# Unmet Need for Contraception among HIV-Positive Women Attending HIV Care and Treatment Service at Saint Paul's Hospital Millennium Medical College, Addis Ababa, Ethiopia

**DOI:** 10.1155/2019/3276780

**Published:** 2019-08-26

**Authors:** Ferid A. Abubeker, Malede B. Fanta, Vanessa K. Dalton

**Affiliations:** ^1^Department of Obstetrics and Gynecology, Saint Paul's Hospital Millennium Medical College, Addis Ababa, Ethiopia; ^2^Program on Women's Healthcare Effectiveness Research, Department of Obstetrics and Gynecology, University of Michigan, Ann Arbor, USA

## Abstract

**Background:**

The emergence of the HIV epidemic is one of the biggest public health challenges the world has ever seen in recent history. Ethiopia is among the countries most affected by the HIV epidemic. The national estimate for the HIV-positive pregnant women was 24,000 for the year 2016, and there were an estimated 3,800 new HIV infections among children. Regardless of their HIV status contraception offers women, their families, and communities a variety of benefits. For HIV-positive women who do not want to become pregnant, contraception has the added benefit of reducing HIV-positive births. Despite its demonstrable contribution, far less attention has been given to prevention of unintended pregnancy as a strategy to PMTCT.

**Objectives:**

To determine the level and contributing factors of unmet need for contraception among HIV-positive women in the ART clinic of Saint Paul's Hospital Millennium Medical College (SPHMMC).

**Methods:**

A facility based cross-sectional study was conducted from 1 September 2016 to 30 November 2016. An exit interview of sampled women enrolled at ART clinic of SPHMMC was done using structured and pretested questionnaire. Descriptive, bivariate, and multivariate methods were used to analyze the level of unmet need and its contributing factors.

**Results:**

The overall unmet need for contraception was 25.1%. The most common reasons for nonuse were related to perceived low risk of pregnancy. Unmet need was more common in unmarried women and those who did not discuss about contraception with HIV care provider. Making joint decision on contraceptive utilization with partner and having serodiscordant partner were associated with decreased odds of unmet need.

**Conclusion:**

The ART clinic represented one of the missed opportunities to initiate and promote contraceptive use. The study also shows broader demand for contraception and the need for new strategies to address the contraceptive needs among HIV-positive clients.

## 1. Introduction 

The emergence of the HIV epidemic is one of the biggest public health challenges the world has ever seen in recent history. Globally, an estimated 36.7 (34.0–39.8) million people were living with HIV in 2015 and there were 2.1 (1.8–2.4) million new HIV infections [1]. Sub-Saharan Africa, home to 70% of all new HIV infections in 2015, is at the epicenter of the HIV epidemic and continues to carry the full brunt of its health and socioeconomic impact [2]. In sub-Saharan Africa, adolescent girls and young women accounted for 25% of new HIV infections among adults, and women accounted for 56% of new HIV infections among adults [1].

Likewise, Ethiopia is among the countries most affected by the HIV epidemic. According to the Ethiopian Demographic Health Survey (EDHS) conducted in 2016, the national adult HIV prevalence was 0.9% with women disproportionately infected, 1.2% compared to 0.6% in men [3]. The country has a large number of people living with HIV (approximately 700,000) and about 500,000 AIDS orphans. The national estimate for the HIV-positive pregnant women was 24,000 for the year 2016, and there were an estimated 3,800 new HIV infections among children in the same year. The coverage of pregnant women who receive ART for PMTCT is just at 69% (50-87) [4].

From a reproductive health- and rights-based perspective, all women should have access to methods that allow them to avoid unintended pregnancies. Regardless of their HIV status contraception offers women, their families, and their communities a variety of benefits. By delaying first births, lengthening birth intervals, reducing the total number of children born to a woman, preventing unintended pregnancies, and reducing the need for unsafe abortions, contraception can have a major impact on improving overall maternal and infant health [5].

For HIV-positive women who do not want to become pregnant, contraception has the added benefit of reducing HIV-positive births and, by extension, the number of children needing HIV-related services. The potential contribution of contraception to preventing HIV-positive births is well established [6, 7]. One study found that even modest decreases in the number of pregnancies to HIV infected women ranging from 6 percent to 35 percent could avert HIV-positive births at the same rates as the use of ARTs for PMTCT [7]. In fact prevention of unintended pregnancy has been one of the WHO four-pronged strategy for PMTCT [8].

Ethiopia has adopted the WHO 4-pronged PMTCT strategy as a key entry point to HIV care for women, men, and families. The Ethiopian ART guidelines do provide guidance on integrated contraceptive/HIV counseling services for HIV-positive women at all levels of care. It also recommends that HIV-positive women should be encouraged to use dual method use (using two forms of contraception, one of which should be a condom) [9]. Despite its demonstrable contribution, far less attention has been given to prevention of unintended pregnancy as a strategy to PMTCT. There are little research incites and substantive data is not available on the magnitude of unintended pregnancy and unmet need of contraception among HIV-positive woman in Ethiopia. Thus, the objective of the study is to determine the level and contributing factors of unmet need for contraception among HIV-positive women.

## 2. Methods

### 2.1. Study Design and Setting

A facility based cross-sectional study was conducted in the ART clinic of Saint Paul's Hospital Millennium Medical College (SPHMMC), Addis Ababa, Ethiopia. Quantitative data was collected from 01 September 2016 to 30 November 2016 through an exit interview.

### 2.2. Study Population

The study population was sampled HIV-positive women of reproductive age (15-49 years) who are married or in union attending the ART clinic and fulfill the eligibility criteria.

### 2.3. Inclusion Criteria

- HIV-positive women who are enrolled in HIV/AIDS care and treatment at time of data collection and are in the reproductive age group (aged between 15 and 49 years).
- Those who are competent to give informed consent.

### 2.4. Exclusion Criteria

- Women who are not married or in union.
- HIV-positive women who are coming for the first time to the ART clinic.

### 2.5. Sample Size and Sampling

Determination of the sample size was according to a previous study done in Johannesburg, South Africa, among HIV-positive women, considering the prevalence of unmet need for contraception to be 29% [10]. A margin of error of 5% (*α*= 0.05) and confidence interval of 95% (standard value of 1.96) were used to give a sample size of 316. To compensate for nonresponse rate 10% of the sample was added. Finally a total of 348 women were sampled for the study. Those who fulfill the inclusion criteria were selected consecutively until the sample size was fulfilled.

### 2.6. Data Collection and Statistical Analysis

Data was collected by four trained data collectors using structured and pretested questionnaire that was translated into Amharic language. The tool consists of sociodemographic characteristics, information on contraceptive utilization, discussion of contraceptive options with ART clinic care provider, and disclosure of HIV infection to partner and partner's HIV status. The tool also includes questions and filters needed to identify woman with unmet need as defined in the DHS analytical studies [11].

Data was analyzed using SPSS version 20.0 for Windows. Descriptive analysis was used to describe the data. Bivariate and multivariate logistic regression analysis of possible explanatory variables over unmet need for contraception was carried out. Bivariate analyses were run and odds ratios were computed to assess the statistical significance of associations;* p*<0.05 was considered significant.

**Figure 1:**
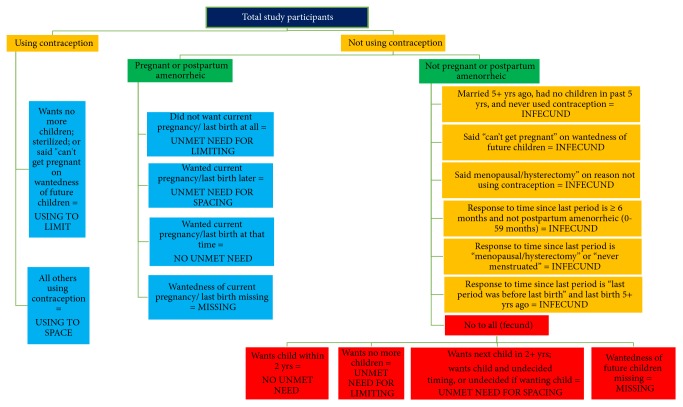
Revised definition of unmet need, currently married women, 2012.

The most recent and standard definition of unmet need published in 2012 by the DHS analytical studies was used to define unmet need [11] Figure 1.

Unmet need was defined as the percentage of all fecund women who are married or in union and not using a method of contraception even though they do not want to get pregnant. Unmet need for spacing includes pregnant women whose pregnancy was mistimed and fecund women who are neither pregnant nor amenorrheic and who are not using any method of contraception and say they want to wait two or more years before their next birth. Unmet need for limiting refers to pregnant women whose pregnancy was unwanted, and fecund women who are neither pregnant nor amenorrheic, who are not using any method of contraception, and who want no more children. Total demand for contraception refers to women with unmet need plus the percentage of currently using contraception (representing “met need”). Proportion of demand satisfied was calculated as percentage of women using contraception divided by the percentage of women with demand for contraception whereas the proportion of demand satisfied by modern methods was calculated as the percentage of women using modern contraception divided by the percentage of women with demand for contraception [11]. Modern methods include male and female sterilization, injectable, intrauterine devices (IUDs), contraceptive pills, implants, female and male condoms, lactational amenorrhoea method, and emergency contraception [12]. Dual method use refers to simultaneous use of two forms of contraception, one of which should be a condom [9].

### 2.7. Ethical Consideration

Prior to data collection ethical review and approval was obtained from the Institutional Review Board (IRB) of the hospital (Ref No. P.M 23/26). Informed written consent was obtained from all participants. Because of the sensitivity of the subject, the names of clients and health care providers were not included in the data collection.

## 3. Results

### 3.1. Demographic and Socioeconomic Characteristics of Participants

A total of 334 out of 348 agreed to participate, giving a response rate of 95.9%. The mean age of respondents was 31.3 years (SD ± 6.2) (Table 1).

### 3.2. Contraceptive Counseling and Utilization

Desire for children was discussed with HIV care provider among 198 (59.3%) of study participants whereas only 166 (49.7%) discussed the use of a contraceptive method with the HIV care provider at the ART clinic.

Among the 112 contraceptive users the most commonly used methods were implants (33%) and injectables (22.3%). Male condom was used by 20.5% whereas pills were used by 13.4% of the study participants. Utilization of IUD and female sterilization was low at 6.3% and 2.7%, respectively. Only 2 (1.8%) women used periodic abstinence constituting for all the traditional contraceptive methods. Thus, the proportion of demand satisfied by modern methods was found to be 56.1%.

### 3.3. Unmet Need for Contraception

**Figure 2:**
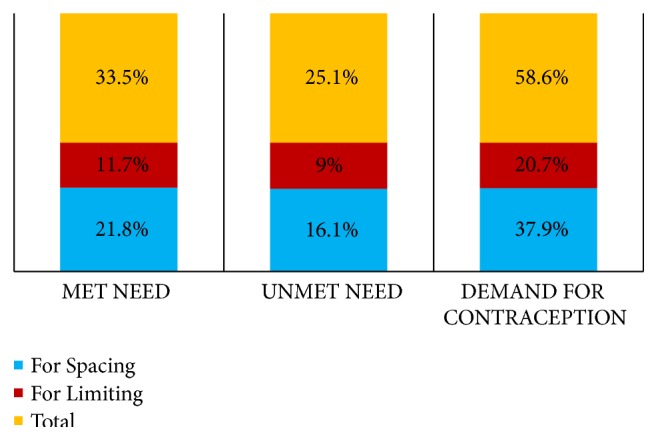
Percentage of met need, unmet need, and demand for contraception among respondents, Addis Ababa, Ethiopia, 2016.

The overall unmet need for contraception was 25.1% of which 16.1% was for spacing and 9% for limiting (Figure 2). The contraceptive utilization (“met need”) was 33.5%. The total demand for contraception was calculated to be 58.6% (37.9% had demand for spacing and 20.7% for limiting). The proportion of demand satisfied was found to be 57.1%.

The most common reasons for nonuse among those with unmet need were exposure related resulting in perceived low risk of pregnancy. These include infrequent sex and not being married (Table 2).

Among the 112 contraceptive users 55 (49.1%) were using dual method. Thus, the demand satisfied for dual method was only 28%.

### 3.4. Determinants of Unmet Need

The logistic regression model showed that women who are living with a man were more likely to have unmet need compared to those who are married (AOR=3.40, 95% CI: 1.34-8.58). Furthermore, not discussing about contraception with HIV care provider and making joint decision on contraceptive use with husband/partner were found to be associated with unmet need (Table 3).

Multivariate logistic regression was also done to identify independent predictors of dual method use. Dual method use was more likely among serodiscordant couples (AOR=6.25, 95% CI: 2.43-16.7) and those who made a joint decision on contraceptive use (AOR=2.94, 95% CI: 1.42-6.25). In contrast, women who did not have their partner support on contraceptive use were less likely to be on dual methods (AOR=3.83, 95% CI: 1.38-10.6).

## 4. Discussion

This study has assessed the level of unmet need for contraception among sampled HIV-positive women of reproductive age attending ART clinic at SPHMMC, Addis Ababa, Ethiopia. Findings suggest that women who did not discuss about contraception with HIV care provider had increased odds of unmet need compared to those who had such discussion. However, less than half discussed about contraception with HIV care provider. Furthermore, neither having such discussion nor being counseled on condom utilization at the ART unit of the hospital improved the use of dual contraceptive methods. Such lack of association may be due to inappropriate contraceptive counseling in the context of HIV/AIDS.

This finding underlines the importance of integrating family planning and reproductive health services into HIV care and treatment services in order to increase family planning uptake and reduce stigma among HIV-positive clients as opposed to free standing contraceptive services. It will also create an opportunity to discuss about sex and fertility desires between HIV positive women and health care workers who already had an ongoing relationship with clients unlike unfamiliar health facilities where women might fear discrimination [13]. A study that looked at HIV-positive men's experiences with integrated family planning (FP) and HIV services in Kenya demonstrated integrated FP/HIV services fostered male inclusion in FP decision making. Furthermore, men appeared invested in FP and their inclusion in FP decision making may bolster both female and male agency. Men's positive attitudes towards FP being provided at HIV care clinics support the programmatic push towards integrated delivery models for FP and HIV services [14].

Though the overall unmet need for contraception among HIV-positive women is similar to the national figure of 22%, it is significantly higher than the 11% unmet need of the general population in Addis Ababa according to the EDHS 2016 [12]. This shows broader demand for contraception and the need for new strategies to address the contraceptive need among HIV-positive clients. Women in our study that were living with a man but not married were at increased odds of having unmet contraceptive needs as compared to currently married women. This finding was consistent with studies done in Zambia and Swaziland where the odds of unmet need were twice among cohabiting couples [15]. Similarly, a study done in Uganda among HIV-positive women showed unmet need was found to be higher in those who are cohabiting without being married compared to those who are married (OR=2.36, 95% CI: 1.16-4.80 [16]. This result may be explained by the misconception that unmarried women and their partners were particularly guarded against pregnancy because they are not in a formal union. The most frequent stated reasons for nonuse among women with unmet need were mainly related to a perception that they had low risk of pregnancy due to infrequent sexual activity and not being married. Appropriate counseling that explores beliefs about risk of pregnancy and addresses misconceptions could therefore improve contraceptive use.

Women who made joint decision with their partner on whether or not to use a contraceptive method were less likely to have unmet need. This result highlights the benefit of active male involvement in reproductive decision making which can further be enhanced by integration of family planning services with HIV care and treatment services where HIV-positive male partners will be visiting routinely compared to stand alone family planning units which are presumed to be visited by female clients only.

Current levels of contraceptive use in all of sub-Saharan Africa are already preventing 173,000 HIV-positive births annually, even though contraception is not widely available in the region. An additional 160,000 HIV-positive births could be averted every year if all women in the region who did not wish to get pregnant could get access to contraceptive services [17].

Modeling studies have demonstrated that mother-to-child transmission of HIV cannot be eliminated without reducing unintended pregnancies among women living with HIV [18].

Findings from this study indicate how significant male partner involvement is in addressing contraceptive use among HIV-positive women. This is especially relevant regarding dual method use as it was reflected in our study by the improved use of dual protection among those who made joint decision on contraceptive use. This calls for strategies that focus on men as well as women. Encouraging communication between couples and increasing male partner involvement through a renewed focus on couples counseling are key in addressing unmet need in general and dual method use in particular.

Though male involvement can have a positive impact, some studies have suggested that male opinions and desires can dictate reproductive health decisions [19]. Thus, providers should give due emphasis to the need of the woman and ensure that efforts to include male partner involvement in FP decision making do not undermine women's reproductive health right and autonomy.

Limitations of the present study include its cross-sectional design. In addition, the study includes more sensitive issues. Thus, social desirability bias could not be ruled out. Consequently, some women accessing the ART services may report a higher use of condom and other contraceptive methods. This can overestimate contraceptive utilization rate. Similarly women may report a birth or current pregnancy as wanted once the child is born. This, on the other hand, can result in an underestimation of the true extent of unwanted births and, therefore, unmet need. Furthermore, the study was conducted in a single teaching hospital in the capital of the nation which limits generalizability of the findings to other settings.

## 5. Conclusion 

The ART clinic represented one of the missed opportunities to initiate and promote contraceptive use. Appropriate counseling that explores beliefs about risk of pregnancy and addresses misconceptions could improve contraceptive use. The study also shows broader demand for contraception and the need for new strategies to address the contraceptive needs among HIV-positive clients.

## Figures and Tables

**Table 1:** Demographic and socioeconomic characteristics of respondents, Addis Ababa, Ethiopia, 2016.

Characteristics	Frequency (N)	Percentage (%)
*Age (Years)*		
15-19	6	1.8
20-24	36	10.8
25-29	88	26.3
30-34	74	22.1
35-39	71	21.3
40-44	41	12.3
45-49	18	5.4
*Current Marital Status*		
Currently Married	193	57.8
Unmarried but living with a man	141	42.2
*Educational Level*		
No education	78	23.4
Primary	71	21.2
Secondary	92	27.5
Technical/Vocational	41	12.3
Higher	52	15.6
*Occupation *		
Student	21	6.3
Unemployed	69	20.6
Housewife	77	23.0
Daily laborer	37	11.1
Government employee	45	13.5
Private employee	72	21.6
Commercial sex worker	13	3.9
*Monthly Income (Ethiopian Birr)*		
None	166	49.7
<500	30	9.0
501-1500	70	20.9
1501-2500	38	11.4
>2501	30	9.0

**Table 2:** Reasons for nonuse of a contraception method in those with unmet need, Addis Ababa, Ethiopia, 2016.

Reason for nonuse	Frequency (N)	Percentage (%)
*Perceived low risk of pregnancy *		
Infrequent sex	16	19.0
Not married	10	11.9
Up to God/fatalistic	7	8.3
Not having sex	4	4.8
*Opposition to use*		
Respondent opposed	9	10.7
Religious prohibition	9	10.7
Partner opposed	7	8.3
*Method related reasons*		
Side effect/health concern	12	14.3
Preferred method not available	6	7.2
Cost too much	4	4.8

*Total*	*84*	*100*

**Table 3:** Association of variables with unmet need for contraception, Addis Ababa, Ethiopia, 2016.

Characteristics	Unmet Need		Crude OR	Adjusted OR
	Yes	No	(95% CI)	(95% CI)
*Age (Years)*				
15-24	24	8	3.62 (1.38-9.52)	2.25 (0.49-10.4)
25-34	36	75	0.58 (0.3-1.13)	0.29 (0.10-0.80)
35-44	24	29	1.00	1.00
*Current Marital Status*				
Currently Married	40	75	1.00	1.00
Unmarried but living with a man	44	37	2.23 (1.24-3.99)^a^	3.40 (1.34-8.55)^a^
*Educational Level*				
No education	10	23	0.39 (0.14-1.04)	0.30 (0.07-1.37)
Primary	15	29	0.46 (0.18-1.14)	0.44 (0.11-1.72)
Secondary	23	33	0.62 (0.27-1.45)	0.85 (0.24-2.95)
Technical/Vocational	17	10	1.52 (0.54-4.21)	1.93 (0.4-9.21)
Higher	19	17	1.00	1.00
*Started ART*				
Yes	68	99	1.00	1.00
No	16	13	1.79 (0.81-3.96)	0.40 (0.09-1.77)
*HIV care provider talked about desire for children*				
Yes	50	80	1.00	1.00
No	24	17	2.26 (1.10-4.61)^a^	1.22 (0.35-4.28)
I don't remember	10	15	1.06 (0.44-2.55)	0.60 (0.10-3.40)
*HIV care provider talked about contraception*				
Yes	39	80	1.00	1.00
No	27	18	3.08 (1.51-6.25)^a^	4.73 (1.36-16.4)^a^
I don't remember	18	14	2.63 (1.18-5.84)^a^	3.63 (1.08-12.2)^a^
*Decision to use or not to use contraception *				
Mainly respondent	54	40	1.00	1.00
Mainly husband/partner	8	4	1.48 (0.41-5.26)	0.94 (0.17-5.18)
Joint decision	22	68	0.24 (0.12-0.45)^a^	0.26 (0.10-0.64)^a^
*Partner HIV status*				
Positive	54	59	1.00	1.00
Negative	5	28	0.19 (0.07-0.54)^a^	0.18 (0.06-0.66)^a^
I don't know/ don't want to tell	7	5	1.53 (0.46-5.10)	1.73 (0.39-7.72)

^a^ Statistically significant, p<0.05.

## Data Availability

The datasets supporting the conclusions of this article are all included within the article.
